# A one-dimensional bromide-bridged Pt^II^/Pt^IV^ mixed-valence complex with a 2-bromo­ethane­sulfonate counter-ion

**DOI:** 10.1107/S2056989017009598

**Published:** 2017-07-04

**Authors:** Nobuyuki Matsushita, Ayako Taira, Yoshiya Taoka

**Affiliations:** a Department of Chemistry & Research Center for Smart Molecules, Rikkyo University, Nishi-Ikebukuro 3-34-1, Toshima-ku, 171-8501 Tokyo, Japan; b Department of Chemistry, Graduate School of Arts and Sciences, The University of Tokyo, Komaba 3-8-1, Meguro-ku, 153-8902 Tokyo, Japan; c Department of Chemistry, Rikkyo University, Nishi-Ikebukuro 3-34-1, Toshima-ku, 171-8501 Tokyo, Japan

**Keywords:** crystal structure, platinum complex, one-dimensional chain complex, halide-bridged complex, *MX*-chain structure, Pt^II,IV^ mixed valence

## Abstract

The title salt, *catena*-poly[[[bis­(ethyl­enedi­amine)­platinum(II)]-μ-bromido-[bis(ethyl­enedi­amine)­platinum(IV)]-μ-bromido] tetra­kis­(2-bromo­ethane­sulfon­ate) dihydrate], {[Pt^II^Pt^IV^Br_2_(C_2_H_8_N_2_)_4_](C_2_H_4_BrSO_3_)_4_·2H_2_O}_*n*_, crystallizes in the space group *P*2_1_2_1_2. It has a linear chain structure extending parallel to the *c* axis, composed of square-planar [Pt(en)_2_]^2+^ and elongated octa­hedral *trans*-[PtBr_2_(en)_2_]^2+^ cations (en is ethyl­enedi­amine) stacked alternately and bridged by the Br atoms. The Pt site of the [Pt^II/IV^(en)_2_] unit is located on a general position. The Br site, which is also located on a general position, is equally disordered over two positions. The Pt and Br atoms form a slight zigzag ⋯Br—Pt^IV^—Br⋯Pt^II^⋯ chain, with Pt^IV^—Br bond lengths of 2.453 (2) and 2.491 (3) Å, Pt^II^⋯Br contacts of 3.069 (2) and 3.032 (3) Å, and Pt^IV^—Br⋯Pt^II^ angles of 178.06 (13) and 177.70 (13)°. The mixed-valence state of the Pt site is expressed by the parameter δ = (Pt^IV^–Br)/(Pt^II^⋯Br), with values of 0.799 and 0.822 for the two independent Br atoms. In the crystal, N—H⋯O and O—H⋯O hydrogen bonds between the amine groups of the Pt complex chains, the sulfonate groups and water mol­ecules of crystallization, stabilize the cationic columnar structure.

## Abstract 2

The characteristic feature in the crystal structure of the title salt is the formation of a columnar chain structure in the cation comprising mixed-valent ⋯Br—Pt^IV^—Br⋯Pt^II^⋯ entities.

## Chemical context   

The title mixed-valence compound, [Pt^II^(en)_2_][Pt^IV^Br_2_(en)_2_](BrC_2_H_4_SO_3_)_4_·2H_2_O (en is ethyl­enedi­amine, C_2_N_2_H_8_), (I), is a member of the family of one-dimensional halogenido-bridged mixed-valence metal complexes, form­ulated as [*M* ^II^(*AA*)_2_][*M* ^IV^ *X*_2_(*AA*)_2_]*Y*_4_ [*M* ^II^/*M* ^IV^ = Pt^II^/Pt^IV^, Pd^II^/Pd^IV^, Ni^II^/Ni^IV^, Pd^II^/Pt^IV^, Ni^II^/Pt^IV^; *X* = Cl, Br, I; *AA* = NH_2_(CH_2_)_2_NH_2_, *etc*.; *Y* = ClO_4_^−^, HSO_4_^−^, *X* ^−^, *etc*.], hereafter abbreviated as *MX*-chain structures, which occur in typical mixed-valence compounds belonging to class II in the classification of Robin & Day (1967 ▸). Compounds with *MX*-chain structures have attracted much inter­est because of their one-dimensional mixed-valence electron systems, as described in a previous report (Matsushita, 2006 ▸).

The metal–halogen distances in compounds with *MX*-chain structures characterize the physical properties based on the mixed-valence electronic state. Compound (I) is one of the first examples of such a compound comprising a sulfonate ion having an alkyl group with a halogen atom at the terminal position, as an organic part of the counter-ion.
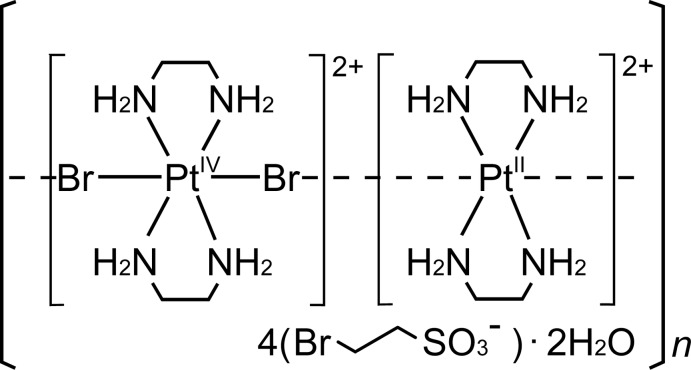


## Structural commentary   

**Figure 1:**
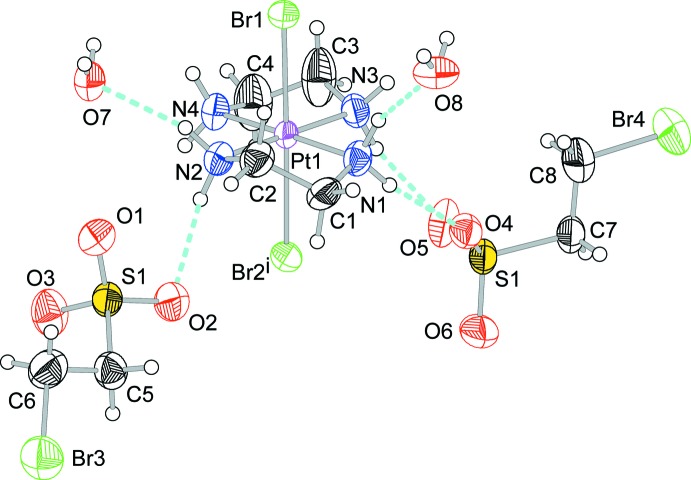
The structures of the mol­ecular components of compound (I), showing the atomic numbering scheme. Displacement ellipsoids are drawn at the 50% probability level for non-H atoms. Light-blue dashed lines represent N—H⋯O hydrogen bonds. Each site of atoms Br1 and Br2 is half occupied. [Symmetry code: (i) *x*, *y*, *z* − 1.]

**Figure 2:**
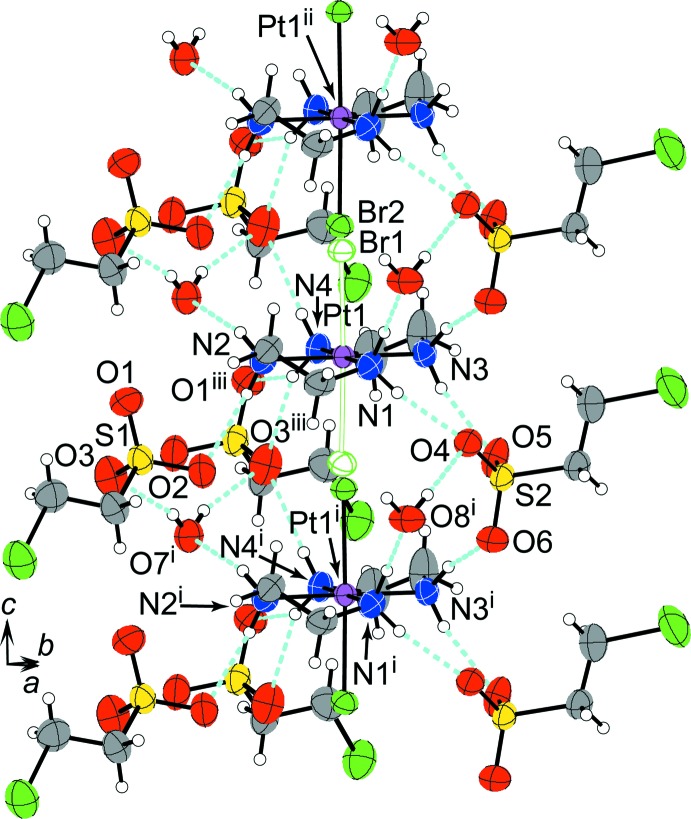
A view of the columnar structure of compound (I), running parallel to the *c* axis. Displacement ellipsoids are drawn at the 50% probability level for non-H atoms. The green hollow Br ellipsoids and the green hollow lines between Pt and Br atoms represent the disordered part of the Pt–Br chain. Light-blue dashed lines represent hydrogen bonds. [Symmetry codes: (i) *x*, *y*, *z* − 1; (ii) *x*, *y*, *z* + 1; (iii) −*x*, −*y* + 1, *z*.]

The structures of the mol­ecular components of (I) are displayed in Fig. 1 ▸. The asymmetric unit of (I) comprises of a Pt-complex moiety, [Pt^II^(en)_2_]^2+^ or [Pt^IV^Br_2_(en)_2_]^2+^, two 2-BrCH_2_CH_2_SO_3_^−^ anions, and two half-mol­ecules of water, the O atoms of which are located on a site with symmetry ..2. The Pt-complex moiety and the sulfonate anions lie on general positions. As shown in Fig. 2 ▸, the structure of (I) is built up of columns extending parallel to the *c* axis, composed of square-planar [Pt(en)_2_]^2+^ and elongated octa­hedral *trans*-[PtBr_2_(en)_2_]^2+^ cations stacked alternately and bridged by the Br atoms. The Pt and Br atoms form an infinite slight zigzag ⋯Br—Pt^IV^—Br⋯Pt^II^⋯ chain. The Br atoms are not located at the exact midpoint between adjacent Pt atoms and are equally disordered over two sites close to the midpoint. Thus, the Pt site is occupationally disordered by Pt^II^ and Pt^IV^ atoms. The valence ordering of the Pt site in (I) belongs to one of three different classes of the order–disorder problem pointed out by Keller (1982 ▸). The structure of (I) can be regarded as being of an one-dimensionally ordered structure type, with the other two directions being in a disordered state. The structural order–disorder situation of the Pt site in (I) has also been observed in the structures of a number of other *MX*-chain compounds (Endres *et al.*, 1980 ▸; Beauchamp *et al.*, 1982 ▸; Cannas *et al.*, 1983 ▸; Yamashita *et al.*, 1985 ▸; Matsushita *et al.*, 1992 ▸; Toriumi *et al.*, 1993 ▸; Huckett *et al.*, 1993 ▸; Matsushita, 2003 ▸, 2005*a* ▸,*b* ▸, 2015 ▸; Matsushita & Taira, 2015 ▸).

With respect to the two sites for the disordered Br atoms, the shorter Pt—Br distances are assigned to Pt^IV^—Br and the longer ones to Pt^II^⋯Br, as follows: Br—Pt^IV^—Br; Pt1—Br1 = 2.453 (2) Å, Pt1—Br2 = 2.491 (3) Å, and Br1—Pt^IV^—Br2 = 178.33 (6)°; Br⋯Pt^II^⋯Br; Pt1⋯Br1 = 3.069 (2) Å, Pt⋯Br2 = 3.032 (3) Å, and Br1⋯Pt^II^⋯Br2 = 178.64 (5)°. Bond angles of the Pt—Br chain are Pt1—Br1⋯Pt1 = 178.06 (13)° and Pt1—Br2⋯Pt1 = 177.70 (13)°. Other bond lengths and angles are given in Table 1 ▸.

The structural parameters indicating the mixed-valence state of the Pt atom, expressed by *δ* = (Pt^IV^–Br)/(Pt^II^⋯Br), are 0.799 and 0.822 for Br1 and Br2, respectively. These values are slightly smaller than those of [Pt(tn)_2_][PtBr_2_(tn)_2_](BF_4_)_4_ (tn is 1,3-di­amino­propane; 0.826; Cannas *et al.*, 1983 ▸), [Pt(en)_2_][PtBr_2_(en)_2_](ClO_4_)_4_ (0.827 for a higher temperature phase at 313 K exhibiting space-group type *Ibam* and 0.828 for a lower temperature phase at 298 K exhibiting space-group type *P*2_1_/*m*; Toriumi *et al.*, 1993 ▸), and comparable with those of [Pt(NH_3_)_4_][PtBr_2_(NH_3_)_4_](HSO_4_)_4_ (0.817; Tanaka *et al.*, 1982 ▸), [Pt(tn)_2_][PtBr_2_(tn)_2_](ClO_4_)_4_ (0.815; Cannas *et al.*, 1983 ▸), [Pt(en)_2_][PtBr_2_(en)_2_](HSO_4_)_4_ (0.813; Matsushita *et al.*, 1992 ▸) but larger than those of [Pt(CH_3_CH_2_NH_2_)_4_][PtBr_2_(CH_3_CH_2_NH_2_)_4_]Br_4_ (0.787 and 0.599; Endres *et al.*, 1980 ▸).

## Supra­molecular features   

Hydrogen-bonding inter­actions in (I) (Table 2 ▸) stabilize the columnar structure composed only of the cationic complexes, as shown in Fig. 2 ▸. A [Pt^II/IV^(en)_2_] unit is bound to an adjacent Pt-complex unit in the column by four hydrogen-bond linkages as follows: N1—H1*A*⋯O4⋯H8–O8⋯H1*B*—N1, N2—H2*A*⋯O7—H7⋯O3—S1—O2⋯H2*B*—N2, N3—H3*A*⋯O6—S2—O5⋯H3*B*—N3, N4—H4*A*⋯O3⋯H4*B*—N4. In addition, the donor N4—H4*A* group is also hydrogen bonded to atom O1, and forms a three-centre hydrogen bond. Such hydrogen-bond linkages are a common structural motif in *MX*-chain compounds (Matsushita, 2003 ▸, 2005*a* ▸,*b* ▸, 2006 ▸, 2015 ▸; Matsushita & Taira, 2015 ▸).

**Figure 3:**
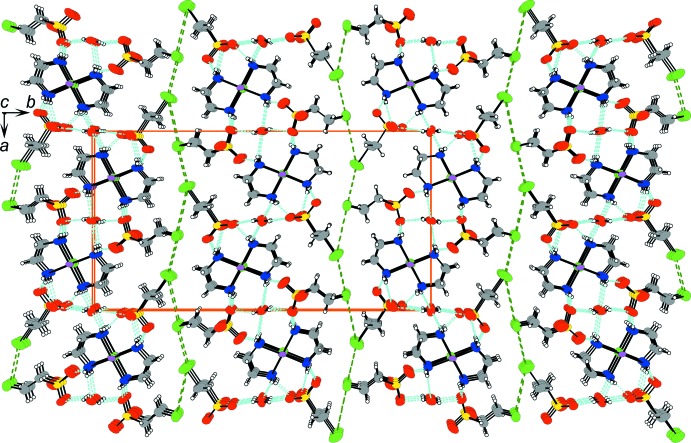
The crystal packing of compound (I), viewed along the *c* axis. Light-blue and green dashed lines represent the hydrogen bonds and the short contacts between Br atoms. Orange solid lines indicate the unit cell.

The columns are arranged in layers parallel to the *ac* plane as a result of the inter­columnar hydrogen-bond linkages, connecting in the direction of the *a* axis, as shown in Figs. 3 ▸ and 4 ▸. Stacking the layers to the direction of the *b* axis makes the three-dimensional crystal packing through contacts between the terminal Br atoms of the 2-bromo­ethane-1-sulfonate ions. The needle-like crystal form, its elongated direction being parallel to the *c* axis, does not reflect the layer structure but the columnar structure. The crystal form suggests that the Br⋯Br contacts contribute as much to binding the layers and constructing the crystal packing as the inter­columnar hydrogen-bond linkages. Such terminal Br atoms of the alkyl chain therefore appear as significant contributors to the crystal packing.

## Synthesis and crystallization   

The title compound was prepared by a procedure similar to a previous literature protocal (Matsushita & Taira, 1999 ▸). To a solution of [Pt(en)_2_]Cl_2_ (0.231 g, 0.598 mmol) solved in a mixture of water (10 ml) and ethanol (2 ml) was added an ethano­lic solution (2 ml) of Br_2_ (32 µl, 0.62 mmol). After removing excess Br_2_ by heating for 2.5 h, to this solution (including the Pt^IV^ complex species) was added an aqueous solution of [Pt(en)_2_]Cl_2_ (0.346 g, 0.896 mmol), and then an aqueous solution of sodium 2-bromo­ethane­sulfonate (3.414 g, 0.0162 mol). The resulting solution was allowed to stand at room temperature for about one month. Metallic lustrous green needle-like crystals of (I) suitable for X-ray analysis were obtained and were collected by filtration (yield 0.553 g, 0.350 mmol, 59%, based on Pt^IV^).

## Refinement   

Crystal data, data collection and structure refinement details are summarized in Table 3 ▸. The H atoms were placed in geometrically calculated positions and refined as riding, with C—H = 0.97 Å, N—H = 0.89 Å, and O—H = 0.82 Å, and with the constraint *U*_iso_(H) = 1.5*U*_eq_(C,N,O). Reflections (0 2 0) and (1 2 0) were affected by the beam-stop and were omitted in the final refinement. The maximum and minimum electron density peaks are located 0.25 and 0.77 Å, respectively, from atom Pt1.

## Supplementary Material

Crystal structure: contains datablock(s) global, I. DOI: 10.1107/S2056989017009598/wm5394sup1.cif

Structure factors: contains datablock(s) I. DOI: 10.1107/S2056989017009598/wm5394Isup2.hkl

CCDC reference: 1559067

Additional supporting information:  crystallographic information; 3D view; checkCIF report

## Figures and Tables

**Figure 4:**
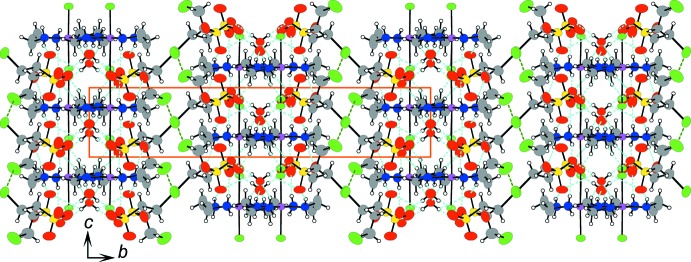
The crystal packing of compound (I), projected on the *bc* plane. Light-blue and green dashed lines represent hydrogen bonds and the short contacts between Br atoms. Orange solid lines indicate the unit cell.

**Table 1:** Selected geometric parameters (Å, °)

Pt1—N2	2.039 (7)	Br3—Br4^ii^	4.429 (2)
Pt1—N1	2.039 (7)	S1—O1	1.425 (9)
Pt1—N3	2.040 (7)	S1—O3	1.442 (8)
Pt1—N4	2.046 (8)	S1—O2	1.444 (8)
N1—C1	1.490 (13)	S1—C5	1.802 (10)
N2—C2	1.498 (13)	C5—C6	1.493 (15)
N3—C3	1.494 (15)	Br4—C8	1.940 (12)
N4—C4	1.511 (14)	S2—O5	1.442 (8)
C1—C2	1.494 (14)	S2—O6	1.459 (9)
C3—C4	1.387 (19)	S2—O4	1.464 (7)
Br3—C6	1.970 (12)	S2—C7	1.789 (10)
Br3—Br4^i^	3.822 (2)	C7—C8	1.461 (18)
			
N2—Pt1—N1	83.6 (3)	C3—C4—N4	113.7 (11)
N3—Pt1—N4	84.1 (3)	O1—S1—O3	110.6 (6)
N2—Pt1—Br1	89.2 (3)	O1—S1—O2	113.7 (6)
N1—Pt1—Br1	91.3 (3)	O3—S1—O2	111.8 (6)
N3—Pt1—Br1	90.9 (3)	O1—S1—C5	109.4 (6)
N4—Pt1—Br1	89.6 (3)	O3—S1—C5	105.8 (5)
N2—Pt1—Br2	89.1 (3)	O2—S1—C5	104.9 (5)
N1—Pt1—Br2	92.8 (3)	C6—C5—S1	108.7 (8)
N3—Pt1—Br2	90.9 (3)	C5—C6—Br3	107.7 (8)
N4—Pt1—Br2	88.2 (3)	O5—S2—O6	112.0 (6)
C1—N1—Pt1	109.5 (6)	O5—S2—O4	112.2 (5)
C2—N2—Pt1	108.2 (6)	O6—S2—O4	112.9 (5)
C3—N3—Pt1	109.2 (6)	O5—S2—C7	105.9 (5)
C4—N4—Pt1	107.7 (6)	O6—S2—C7	106.3 (5)
N1—C1—C2	107.3 (8)	O4—S2—C7	106.9 (5)
C1—C2—N2	107.9 (8)	C8—C7—S2	109.6 (8)
C4—C3—N3	111.6 (11)	C7—C8—Br4	112.3 (9)

Symmetry codes: (i) 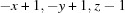; (ii) 
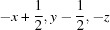
.

**Table 2:** Hydrogen-bond geometry (Å, °)

*D*—H⋯*A*	*D*—H	H⋯*A*	*D*⋯*A*	*D*—H⋯*A*
N1—H1*A*⋯O4	0.89	2.02	2.897 (11)	169
N1—H1*B*⋯O8	0.89	2.16	2.936 (11)	146
N2—H2*A*⋯O7	0.89	2.20	3.022 (9)	153
N2—H2*B*⋯O2	0.89	2.17	2.981 (12)	152
N3—H3*A*⋯O6^iii^	0.89	2.14	2.962 (12)	152
N3—H3*B*⋯O5	0.89	2.06	2.934 (12)	167
N4—H4*A*⋯O1^iv^	0.89	2.48	3.039 (13)	121
N4—H4*A*⋯O3^iv^	0.89	2.36	3.182 (14)	153
N4—H4*B*⋯O3^v^	0.89	2.30	3.186 (13)	172
O7—H7⋯O3^iii^	0.83	2.07	2.874 (11)	161
O8—H8⋯O4^vi^	0.82	2.00	2.811 (10)	169

Symmetry codes: (iii) 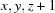; (iv) 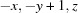; (v) 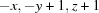; (vi) 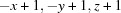.

**Table 3:** Experimental details

Crystal data	
Chemical formula	[Pt(C_2_H_8_N_2_)_2_][PtBr_2_(C_2_H_8_N_2_)_2_](BrC_2_H_4_SO_3_)_4_·2H_2_O
*M*_r_	1578.53
Crystal system, space group	Orthorhombic, *P*2_1_2_1_2
Temperature (K)	296
*a*, *b*, *c* (Å)	14.3568 (8), 27.0628 (13), 5.5212 (2)
*V* (Å^3^)	2145.18 (18)
*Z*	2
Radiation type	Mo *K*α
μ (mm^−1^)	12.36
Crystal size (mm)	0.27 × 0.13 × 0.06

Data collection	
Diffractometer	Rigaku R-AXIS RAPID imaging-plate
Absorption correction	Multi-scan (*ABSCOR*; Higashi, 1995 ▸)
*T*_min_, *T*_max_	0.268, 1.000
No. of measured, independent and observed [*I* > 2σ(*I*)] reflections	48781, 7690, 6041
*R*_int_	0.072
(sin θ/λ)_max_ (Å^−1^)	0.757

Refinement	
*R*[*F* ^2^ > 2σ(*F* ^2^)], *wR*(*F* ^2^), *S*	0.042, 0.103, 1.03
No. of reflections	7690
No. of parameters	238
H-atom treatment	H-atom parameters constrained
Δρ_max_, Δρ_min_ (e Å^−3^)	1.40, −2.29
Absolute structure	Refined as an inversion twin.
Absolute structure parameter	0.081 (14)

Computer programs: *RAPID-AUTO* (Rigaku, 2000 ▸), *SHELXT* (Sheldrick, 2015*a* ▸), *DIAMOND* (Brandenburg, 2017 ▸), *SHELXL2014* (Sheldrick, 2015*b* ▸) and *publCIF* (Westrip, 2010 ▸).

## supplementary crystallographic information

### Crystal data

**Table:**

[Pt(C_2_H_8_N_2_)_2_][PtBr_2_(C_2_H_8_N_2_)_2_](BrC_2_H_4_SO_3_)_4_·2H_2_O	*D*_x_ = 2.444 Mg m^−^^3^
*M**_r_* = 1578.53	Mo *K*α radiation, λ = 0.71069 Å
Orthorhombic, *P*2_1_2_1_2	Cell parameters from 41554 reflections
*a* = 14.3568 (8) Å	θ = 1.4–32.6°
*b* = 27.0628 (13) Å	µ = 12.36 mm^−^^1^
*c* = 5.5212 (2) Å	*T* = 296 K
*V* = 2145.18 (18) Å^3^	Needle, green metallic
*Z* = 2	0.27 × 0.13 × 0.06 mm
*F*(000) = 1492	

### Data collection

**Table:**

Rigaku R-AXIS RAPID imaging-plate diffractometer	7690 independent reflections
Radiation source: X-ray sealed tube	6041 reflections with *I* > 2σ(*I*)
Graphite monochromator	*R*_int_ = 0.072
Detector resolution: 10.00 pixels mm^-1^	θ_max_ = 32.6°, θ_min_ = 1.6°
ω scans	*h* = −21→21
Absorption correction: multi-scan (*ABSCOR*; Higashi, 1995)	*k* = −40→40
*T*_min_ = 0.268, *T*_max_ = 1.000	*l* = −8→7
48781 measured reflections	

### Refinement

**Table:**

Refinement on *F*^2^	Hydrogen site location: inferred from neighbouring sites
Least-squares matrix: full	H-atom parameters constrained
*R*[*F*^2^ > 2σ(*F*^2^)] = 0.042	*w* = 1/[σ^2^(*F*_o_^2^) + (0.0502*P*)^2^ + 1.7628*P*] where *P* = (*F*_o_^2^ + 2*F*_c_^2^)/3
*wR*(*F*^2^) = 0.103	(Δ/σ)_max_ = 0.002
*S* = 1.03	Δρ_max_ = 1.40 e Å^−^^3^
7690 reflections	Δρ_min_ = −2.29 e Å^−^^3^
238 parameters	Extinction correction: SHELXL2014 (Sheldrick, 2015b), Fc^*^=kFc[1+0.001xFc^2^λ^3^/sin(2θ)]^-1/4^
0 restraints	Extinction coefficient: 0.0011 (2)
Primary atom site location: structure-invariant direct methods	Absolute structure: Refined as an inversion twin.
Secondary atom site location: difference Fourier map	Absolute structure parameter: 0.081 (14)

### Special details

**Table:**

Geometry. All esds (except the esd in the dihedral angle between two l.s. planes) are estimated using the full covariance matrix. The cell esds are taken into account individually in the estimation of esds in distances, angles and torsion angles; correlations between esds in cell parameters are only used when they are defined by crystal symmetry. An approximate (isotropic) treatment of cell esds is used for estimating esds involving l.s. planes.
Refinement. Refined as a 2-component inversion twin.

### Fractional atomic coordinates and isotropic or equivalent isotropic displacement parameters (Å^2^)

**Table:**

	*x*	*y*	*z*	*U*_iso_*/*U*_eq_	Occ. (<1)
Pt1	0.24017 (2)	0.56156 (2)	0.28907 (6)	0.03200 (10)	
Br1	0.2416 (2)	0.56002 (11)	0.7333 (4)	0.0411 (5)	0.5
Br2	0.2367 (2)	0.56067 (12)	0.8381 (5)	0.0434 (5)	0.5
N1	0.3661 (5)	0.5269 (3)	0.2750 (16)	0.0417 (17)	
H1A	0.4016	0.5409	0.1619	0.063*	
H1B	0.3950	0.5298	0.4169	0.063*	
N2	0.1894 (5)	0.4912 (3)	0.2898 (16)	0.0433 (17)	
H2A	0.1439	0.4884	0.3986	0.065*	
H2B	0.1663	0.4838	0.1447	0.065*	
N3	0.2941 (5)	0.6313 (3)	0.2881 (16)	0.0409 (16)	
H3A	0.3394	0.6336	0.3976	0.061*	
H3B	0.3181	0.6381	0.1432	0.061*	
N4	0.1146 (5)	0.5973 (3)	0.2971 (18)	0.0451 (17)	
H4A	0.0776	0.5858	0.1810	0.068*	
H4B	0.0869	0.5925	0.4393	0.068*	
C1	0.3522 (7)	0.4736 (4)	0.217 (2)	0.051 (2)	
H1C	0.4061	0.4546	0.2667	0.077*	
H1D	0.3434	0.4693	0.0442	0.077*	
C2	0.2676 (8)	0.4568 (4)	0.3516 (19)	0.050 (2)	
H2C	0.2793	0.4574	0.5246	0.075*	
H2D	0.2517	0.4233	0.3050	0.075*	
C3	0.2185 (9)	0.6674 (4)	0.346 (4)	0.081 (5)	
H3C	0.2146	0.6716	0.5204	0.121*	
H3D	0.2337	0.6992	0.2756	0.121*	
C4	0.1329 (9)	0.6517 (4)	0.258 (4)	0.082 (5)	
H4C	0.1299	0.6588	0.0863	0.123*	
H4D	0.0841	0.6706	0.3372	0.123*	
Br3	0.08492 (11)	0.25167 (5)	−0.4944 (3)	0.0718 (4)	
S1	0.08693 (18)	0.39688 (9)	−0.1163 (5)	0.0473 (6)	
O1	0.0837 (7)	0.3825 (4)	0.1320 (16)	0.074 (3)	
O2	0.1537 (6)	0.4354 (3)	−0.1664 (17)	0.074 (2)	
O3	−0.0049 (5)	0.4096 (3)	−0.202 (2)	0.067 (2)	
C5	0.1242 (7)	0.3452 (3)	−0.298 (2)	0.052 (2)	
H5A	0.1201	0.3535	−0.4682	0.078*	
H5B	0.1884	0.3371	−0.2606	0.078*	
C6	0.0628 (9)	0.3020 (4)	−0.243 (2)	0.065 (3)	
H6A	−0.0020	0.3121	−0.2443	0.098*	
H6B	0.0774	0.2888	−0.0845	0.098*	
Br4	0.68975 (12)	0.71974 (7)	0.1645 (4)	0.1022 (6)	
S2	0.47517 (16)	0.62794 (9)	−0.2099 (5)	0.0430 (5)	
O4	0.4804 (5)	0.5844 (3)	−0.0529 (13)	0.0478 (16)	
O5	0.3986 (5)	0.6599 (3)	−0.1465 (17)	0.059 (2)	
O6	0.4751 (7)	0.6155 (4)	−0.4668 (15)	0.067 (2)	
C7	0.5787 (8)	0.6632 (4)	−0.158 (2)	0.053 (3)	
H7A	0.6330	0.6430	−0.1912	0.079*	
H7B	0.5799	0.6915	−0.2649	0.079*	
C8	0.5809 (10)	0.6798 (6)	0.094 (3)	0.080 (4)	
H8A	0.5806	0.6512	0.2002	0.120*	
H8B	0.5252	0.6989	0.1278	0.120*	
O7	0.0000	0.5000	0.525 (2)	0.058 (3)	
H7	0.0086	0.4769	0.6227	0.087*	
O8	0.5000	0.5000	0.655 (2)	0.056 (3)	
H8	0.4991	0.4763	0.7486	0.085*	

### Atomic displacement parameters (Å^2^)

**Table:**

	*U*^11^	*U*^22^	*U*^33^	*U*^12^	*U*^13^	*U*^23^
Pt1	0.02252 (13)	0.04414 (15)	0.02934 (15)	−0.00288 (12)	−0.00031 (10)	−0.00007 (11)
Br1	0.0407 (11)	0.0549 (10)	0.0277 (9)	−0.0046 (17)	−0.0016 (14)	0.0015 (14)
Br2	0.0392 (11)	0.0578 (10)	0.0331 (11)	−0.0020 (17)	−0.0062 (12)	0.0006 (14)
N1	0.028 (3)	0.052 (4)	0.045 (4)	0.002 (3)	−0.001 (3)	0.008 (3)
N2	0.033 (4)	0.050 (4)	0.047 (4)	0.001 (3)	0.004 (4)	−0.003 (4)
N3	0.033 (4)	0.046 (4)	0.044 (4)	−0.006 (3)	−0.004 (4)	0.001 (3)
N4	0.031 (4)	0.050 (4)	0.054 (5)	−0.004 (3)	0.003 (4)	0.001 (4)
C1	0.042 (5)	0.067 (6)	0.045 (5)	0.006 (4)	0.010 (5)	−0.005 (5)
C2	0.050 (6)	0.052 (5)	0.048 (5)	−0.004 (4)	0.006 (5)	0.006 (4)
C3	0.045 (7)	0.049 (6)	0.149 (14)	0.002 (5)	0.003 (8)	0.000 (8)
C4	0.052 (7)	0.058 (6)	0.136 (15)	0.009 (5)	0.001 (9)	−0.002 (8)
Br3	0.0743 (10)	0.0543 (6)	0.0867 (9)	0.0003 (6)	0.0018 (7)	−0.0150 (6)
S1	0.0378 (13)	0.0503 (12)	0.0537 (15)	−0.0055 (10)	0.0055 (11)	−0.0100 (10)
O1	0.063 (6)	0.109 (7)	0.050 (5)	−0.022 (5)	0.010 (4)	−0.003 (5)
O2	0.069 (5)	0.083 (5)	0.070 (6)	−0.034 (5)	0.015 (4)	−0.020 (5)
O3	0.045 (4)	0.060 (4)	0.096 (7)	0.010 (3)	−0.006 (5)	−0.007 (5)
C5	0.042 (5)	0.052 (5)	0.063 (7)	0.001 (4)	0.000 (5)	−0.011 (5)
C6	0.062 (7)	0.068 (7)	0.066 (8)	−0.001 (5)	0.013 (6)	−0.007 (6)
Br4	0.0756 (11)	0.1267 (14)	0.1041 (13)	−0.0417 (10)	−0.0052 (9)	−0.0394 (11)
S2	0.0333 (11)	0.0518 (12)	0.0437 (12)	−0.0085 (8)	−0.0021 (11)	0.0087 (11)
O4	0.044 (4)	0.056 (4)	0.043 (4)	−0.004 (3)	−0.006 (3)	0.008 (3)
O5	0.038 (4)	0.052 (4)	0.087 (6)	−0.001 (3)	0.006 (4)	0.018 (4)
O6	0.071 (6)	0.093 (6)	0.038 (4)	−0.023 (5)	−0.005 (4)	0.002 (4)
C7	0.036 (5)	0.074 (7)	0.049 (6)	−0.014 (5)	−0.001 (4)	0.007 (5)
C8	0.061 (9)	0.101 (10)	0.079 (9)	−0.036 (8)	0.000 (8)	−0.004 (8)
O7	0.039 (6)	0.074 (7)	0.061 (8)	−0.008 (5)	0.000	0.000
O8	0.080 (8)	0.049 (5)	0.040 (6)	0.012 (5)	0.000	0.000

### Geometric parameters (Å, º)

**Table:**

Pt1—N2	2.039 (7)	C3—H3C	0.9700
Pt1—N1	2.039 (7)	C3—H3D	0.9700
Pt1—N3	2.040 (7)	C4—H4C	0.9700
Pt1—N4	2.046 (8)	C4—H4D	0.9700
Pt1—Br1	2.453 (2)	Br3—C6	1.970 (12)
Pt1—Br2^i^	2.491 (3)	Br3—Br4^iii^	3.822 (2)
Pt1—Br2	3.032 (3)	Br3—Br4^iv^	4.429 (2)
Pt1—Br1^i^	3.069 (2)	S1—O1	1.425 (9)
Br1—Br2	0.5826 (18)	S1—O3	1.442 (8)
Br1—Pt1^ii^	3.069 (2)	S1—O2	1.444 (8)
Br2—Pt1^ii^	2.491 (3)	S1—C5	1.802 (10)
N1—C1	1.490 (13)	C5—C6	1.493 (15)
N1—H1A	0.8900	C5—H5A	0.9700
N1—H1B	0.8900	C5—H5B	0.9700
N2—C2	1.498 (13)	C6—H6A	0.9700
N2—H2A	0.8900	C6—H6B	0.9700
N2—H2B	0.8900	Br4—C8	1.940 (12)
N3—C3	1.494 (15)	S2—O5	1.442 (8)
N3—H3A	0.8900	S2—O6	1.459 (9)
N3—H3B	0.8900	S2—O4	1.464 (7)
N4—C4	1.511 (14)	S2—C7	1.789 (10)
N4—H4A	0.8900	C7—C8	1.461 (18)
N4—H4B	0.8900	C7—H7A	0.9700
C1—C2	1.494 (14)	C7—H7B	0.9700
C1—H1C	0.9700	C8—H8A	0.9700
C1—H1D	0.9700	C8—H8B	0.9700
C2—H2C	0.9700	O7—H7	0.8350
C2—H2D	0.9700	O8—H8	0.8239
C3—C4	1.387 (19)		
			
N2—Pt1—N1	83.6 (3)	C1—C2—N2	107.9 (8)
N2—Pt1—N3	178.7 (3)	C1—C2—H2C	110.1
N1—Pt1—N3	95.1 (3)	N2—C2—H2C	110.1
N2—Pt1—N4	97.3 (3)	C1—C2—H2D	110.1
N1—Pt1—N4	178.7 (4)	N2—C2—H2D	110.1
N3—Pt1—N4	84.1 (3)	H2C—C2—H2D	108.4
N2—Pt1—Br1	89.2 (3)	C4—C3—N3	111.6 (11)
N1—Pt1—Br1	91.3 (3)	C4—C3—H3C	109.3
N3—Pt1—Br1	90.9 (3)	N3—C3—H3C	109.3
N4—Pt1—Br1	89.6 (3)	C4—C3—H3D	109.3
N2—Pt1—Br2^i^	89.2 (3)	N3—C3—H3D	109.3
N1—Pt1—Br2^i^	88.6 (3)	H3C—C3—H3D	108.0
N3—Pt1—Br2^i^	90.8 (3)	C3—C4—N4	113.7 (11)
N4—Pt1—Br2^i^	90.5 (3)	C3—C4—H4C	108.8
Br1—Pt1—Br2^i^	178.33 (6)	N4—C4—H4C	108.8
N2—Pt1—Br2	89.1 (3)	C3—C4—H4D	108.8
N1—Pt1—Br2	92.8 (3)	N4—C4—H4D	108.8
N3—Pt1—Br2	90.9 (3)	H4C—C4—H4D	107.7
N4—Pt1—Br2	88.2 (3)	C6—Br3—Br4^iii^	110.1 (4)
Br2^i^—Pt1—Br2	177.70 (13)	C6—Br3—Br4^iv^	72.7 (4)
N2—Pt1—Br1^i^	89.5 (3)	Br4^iii^—Br3—Br4^iv^	174.67 (5)
N1—Pt1—Br1^i^	87.1 (3)	O1—S1—O3	110.6 (6)
N3—Pt1—Br1^i^	90.4 (3)	O1—S1—O2	113.7 (6)
N4—Pt1—Br1^i^	91.9 (3)	O3—S1—O2	111.8 (6)
Br1—Pt1—Br1^i^	178.06 (13)	O1—S1—C5	109.4 (6)
Br2—Pt1—Br1^i^	178.64 (5)	O3—S1—C5	105.8 (5)
Br2—Br1—Pt1	172.2 (6)	O2—S1—C5	104.9 (5)
Pt1—Br1—Pt1^ii^	178.06 (13)	C6—C5—S1	108.7 (8)
Br1—Br2—Pt1^ii^	172.0 (6)	C6—C5—H5A	110.0
Pt1^ii^—Br2—Pt1	177.70 (13)	S1—C5—H5A	110.0
C1—N1—Pt1	109.5 (6)	C6—C5—H5B	110.0
C1—N1—H1A	109.8	S1—C5—H5B	110.0
Pt1—N1—H1A	109.8	H5A—C5—H5B	108.3
C1—N1—H1B	109.8	C5—C6—Br3	107.7 (8)
Pt1—N1—H1B	109.8	C5—C6—H6A	110.2
H1A—N1—H1B	108.2	Br3—C6—H6A	110.2
C2—N2—Pt1	108.2 (6)	C5—C6—H6B	110.2
C2—N2—H2A	110.1	Br3—C6—H6B	110.2
Pt1—N2—H2A	110.1	H6A—C6—H6B	108.5
C2—N2—H2B	110.1	O5—S2—O6	112.0 (6)
Pt1—N2—H2B	110.1	O5—S2—O4	112.2 (5)
H2A—N2—H2B	108.4	O6—S2—O4	112.9 (5)
C3—N3—Pt1	109.2 (6)	O5—S2—C7	105.9 (5)
C3—N3—H3A	109.8	O6—S2—C7	106.3 (5)
Pt1—N3—H3A	109.8	O4—S2—C7	106.9 (5)
C3—N3—H3B	109.8	C8—C7—S2	109.6 (8)
Pt1—N3—H3B	109.8	C8—C7—H7A	109.8
H3A—N3—H3B	108.3	S2—C7—H7A	109.8
C4—N4—Pt1	107.7 (6)	C8—C7—H7B	109.8
C4—N4—H4A	110.2	S2—C7—H7B	109.8
Pt1—N4—H4A	110.2	H7A—C7—H7B	108.2
C4—N4—H4B	110.2	C7—C8—Br4	112.3 (9)
Pt1—N4—H4B	110.2	C7—C8—H8A	109.2
H4A—N4—H4B	108.5	Br4—C8—H8A	109.2
N1—C1—C2	107.3 (8)	C7—C8—H8B	109.2
N1—C1—H1C	110.3	Br4—C8—H8B	109.2
C2—C1—H1C	110.3	H8A—C8—H8B	107.9
N1—C1—H1D	110.3	H7—O7—H7^v^	99.4
C2—C1—H1D	110.3	H8—O8—H8^vi^	102.2
H1C—C1—H1D	108.5		

Symmetry codes: (i) *x*, *y*, *z*−1; (ii) *x*, *y*, *z*+1; (iii) −*x*+1, −*y*+1, *z*−1; (iv) −*x*+1/2, *y*−1/2, −*z*; (v) −*x*, −*y*+1, *z*; (vi) −*x*+1, −*y*+1, *z*.

### Hydrogen-bond geometry (Å, º)

**Table:**

*D*—H···*A*	*D*—H	H···*A*	*D*···*A*	*D*—H···*A*
N1—H1*A*···O4	0.89	2.02	2.897 (11)	169
N1—H1*B*···O8	0.89	2.16	2.936 (11)	146
N2—H2*A*···O7	0.89	2.20	3.022 (9)	153
N2—H2*B*···O2	0.89	2.17	2.981 (12)	152
N3—H3*A*···O6^ii^	0.89	2.14	2.962 (12)	152
N3—H3*B*···O5	0.89	2.06	2.934 (12)	167
N4—H4*A*···O1^v^	0.89	2.48	3.039 (13)	121
N4—H4*A*···O3^v^	0.89	2.36	3.182 (14)	153
N4—H4*B*···O3^vii^	0.89	2.30	3.186 (13)	172
O7—H7···O3^ii^	0.83	2.07	2.874 (11)	161
O8—H8···O4^viii^	0.82	2.00	2.811 (10)	169

Symmetry codes: (ii) *x*, *y*, *z*+1; (v) −*x*, −*y*+1, *z*; (vii) −*x*, −*y*+1, *z*+1; (viii) −*x*+1, −*y*+1, *z*+1.
